# Monocytes Latently Infected with Human Cytomegalovirus Evade Neutrophil Killing

**DOI:** 10.1016/j.isci.2019.01.007

**Published:** 2019-01-08

**Authors:** Elizabeth Elder, Benjamin Krishna, James Williamson, Yusuf Aslam, Neda Farahi, Alexander Wood, Veronika Romashova, Kate Roche, Eain Murphy, Edwin Chilvers, Paul J. Lehner, John Sinclair, Emma Poole

**Affiliations:** 1 Department of Medicine, University of Cambridge, Addenbrooke's Hospital, Hills Road, Cambridge CB2 0QQ, UK; 2 Genomic Medicine Institute, Lerner Research Institute, 9620 Carnegie Avenue, Cleveland, OH, USA; 3 Cleveland Clinic, Lerner Research Institute, Cleveland, OH, USA

**Keywords:** Molecular Mechanism of Behavior, Immunology, Immune Response, Virology

## Abstract

One site of latency of human cytomegalovirus (HCMV) *in vivo* is in undifferentiated cells of the myeloid lineage. Although latently infected cells are known to evade host T cell responses by suppression of T cell effector functions, it is not known if they must also evade surveillance by other host immune cells. Here we show that cells latently infected with HCMV can, indeed, be killed by host neutrophils but only in a serum-dependent manner. Specifically, antibodies to the viral latency-associated US28 protein mediate neutrophil killing of latently infected cells. To address this mechanistically, a full proteomic screen was carried out on latently infected monocytes. This showed that latent infection downregulates the neutrophil chemoattractants S100A8/A9, thus suppressing neutrophil recruitment to latently infected cells. The ability of latently infected cells to inhibit neutrophil recruitment represents an immune evasion strategy of this persistent human pathogen, helping to prevent clearance of the latent viral reservoir.

## Highlights

• Neutrophils can target HCMV latently infected monocytes for ADCC-mediated killing
• Killing of latently infected cells by neutrophils requires high E:T ratios
• Latent infection reduces secretion of the neutrophil chemoattractants S100A8/A9
• Decreased S100A8/A9 secretion prevents neutrophil targeting of latently infected cells

## Graphical Abstract

**Figure:**
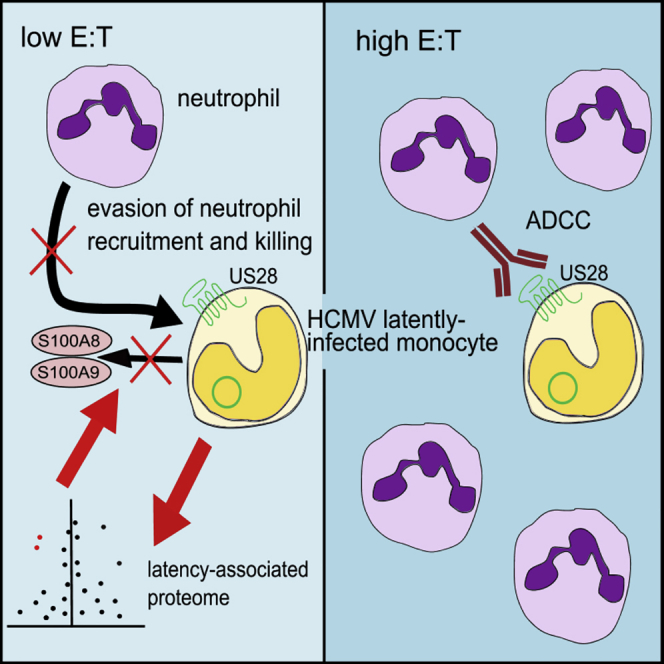

## Introduction

Human cytomegalovirus (HCMV) is a human herpesvirus that can cause severe disease in immune-suppressed, immune-compromised, and immune-naive individuals. For instance, it is the leading viral cause of birth defects in the developed world (Terrazzini and Kern, 2014). There is no effective vaccine against HCMV, and routinely used antivirals suffer from a number of drawbacks including poor bioavailability, toxic side effects, and the risk of emergence of drug resistance (Benzi et al., 2012, Hantz et al., 2010, Komatsu et al., 2010, Scott et al., 2007). One other aspect of HCMV biology also makes antiviral targeting difficult, and this is the ability of the virus to establish a latent infection in certain cell types *in vivo*. During latent infection, the maintenance of viral genome is underpinned by a latency-associated transcription program but in the absence of virus production. This likely helps the virus to avoid immune detection and clearance by host immune responses (Poole et al., 2014a, Poole et al., 2014b, Poole and Sinclair, 2015, Sinclair and Poole, 2014, Wills et al., 2015). However, it is now clear that a substantial level of HCMV disease in immunocompromised subjects results from reactivation of virus from these latently infected cells (Sissons and Wills, 2015).

One site of HCMV latency, *in vivo*, is in the myeloid cells such as CD14+ monocytes and their CD34+ progenitors. As these cells differentiate into dendritic cells (DCs), or macrophages, viral lytic gene expression reactivates leading to viral DNA replication and *de novo* production of infectious virions. Consequently, HCMV lifelong persistence likely results from constant reactivation of the virus from latency, but, in the immune-competent individuals, these reactivation events are kept sub-clinical by normal host immune responses (Poole et al., 2014a, Poole et al., 2014b, Poole and Sinclair, 2015, Sinclair and Poole, 2014, Wills et al., 2015).

Understanding latent carriage is clearly important for a full understanding of how this persistent human pathogen interacts with its host, and, lately, substantial progress has been made in identifying the effects of latent infection on the latently infected cell. For instance, although the transcription program of key lytic genes is heavily repressed during HCMV latency, a number of viral genes are known to be expressed in latently infected myeloid cells (Cheng et al., 2017, Dupont and Reeves, 2016, Shnayder et al., 2018) and the effects of some of these on latently infected cells have been reported (Humby and O'Connor, 2015, Keyes et al., 2013, Lau et al., 2016b, Poole et al., 2014a, Poole et al., 2014b, Weekes et al., 2013). This has uncovered a number of ways by which latency-associated viral gene expression manipulates the cell to optimize carriage and reactivation of latent viral genomes (Mason et al., 2012, Poole and Sinclair, 2015). Importantly, such studies have also led to proof of principals for chemotherapeutic (Krishna et al., 2017b, Weekes et al., 2013) and immunotherapeutic strategies to target the latent reservoir (Krishna et al., 2016) *in vitro*, although whether these will have clinical benefits is still under investigation.

Besides the known changes in the latently infected cell, which can modulate, e.g., cell survival (Krishna et al., 2016, Mason et al., 2012, Poole et al., 2008, Poole et al., 2015, Poole and Sinclair, 2015, Krishna et al., 2017a, Lau et al., 2016b), it is also clear that other latency-associated changes in cellular gene expression can manipulate the cellular microenvironment to also help latently infected cells evade T cell surveillance (Lau et al., 2016b, Mason et al., 2012, Mason et al., 2013). However, whereas there is a wealth of data regarding the effector functions of natural killer cells and neutrophils during lytic infection with HCMV (Bennett et al., 2010, Falk et al., 2002, Heo et al., 2015, Magri et al., 2011, Stern-Ginossar et al., 2008, Wills et al., 2005, Yamin et al., 2016), much less is known about whether these immune cells can detect and target latently infected cells and, if so, how latent infection combats such antiviral functions.

Neutrophils comprise about 50% of all leukocytes. These cells are rapidly recruited to sites of infection or inflammation by chemotaxis. Here, they shape the immune landscape through interactions with macrophages, DCs, and cells of the adaptive immune response by direct cell-cell contact or via soluble mediators (Mocsai, 2013, Moraes et al., 2006, Nani et al., 2015, Peters and Sacks, 2009, Ribeiro-Gomes and Sacks, 2012, Williams and Chambers, 2014). Once recruited, neutrophils become fully activated, characterized by their ability to release granule proteins, their acquisition of phagocytic capabilities, the production of reactive oxygen species (ROS), and their ability to produce neutrophil extracellular traps (NETs), all of which enhance the cells' effector capacity (Brinkmann et al., 2004, Kruger et al., 2015). In addition, neutrophils are able to mediate antigen-dependent cell cytotoxicity (ADCC) via Fc receptors on their cell surface, which allows recognition of pathogen-infected cells and target them for killing (Sionov et al., 2015, Sips et al., 2016, Yu et al., 2016). Recently, neutrophil-mediated ADCC killing has been the subject of intense research with clinical trials in place for using this function of neutrophils to target cancerous cells (Challacombe et al., 2006, Di Carlo et al., 2001a, Di Carlo et al., 2001b, Matlung et al., 2018, Rajasekaran et al., 2015, Treffers et al., 2018). There is also an increasing body of evidence to suggest that neutrophils target virally infected cells (Fujisawa, 2008, Jegaskanda et al., 2013, Worley et al., 2018). Neutrophils have been shown to play antiviral roles via ROS for a number of viruses (Drescher and Bai, 2013), and herpesviruses, including HCMV, have been shown to induce ROS in phagocytic cells during lytic infection (Gonzalez-Dosal et al., 2011, Speir et al., 1996). Similarly, a number of viruses have also been implicated to be targeted by NETs (Saitoh et al., 2012) as well as ADCC (Sionov et al., 2015, Sips et al., 2016, Yu et al., 2016). Indeed, it is known that cells lytically infected with HCMV both induce interleukin (IL)-8 (Costa et al., 2013) and express a virally encoded CXCL1 homolog, UL146 (Luttichau, 2010), which both attracts neutrophils and then uses these attracted immune cells as a “Trojan horse” to help disseminate infectious virions (Pocock et al., 2017). Although it is clear that such a strategy could aid the dissemination of virus from lytically infected cells, this will not occur during a latent infection as no virion production occurs during this phase of the virus life cycle (Hargett and Shenk, 2011, Poole et al., 2014a, Poole et al., 2014b) In addition, previous reports have not identified UL146 as being expressed during latency (Goodrum et al., 2002, Shnayder et al., 2018, Slobedman and Cheung, 2008). Consequently, we have analyzed whether neutrophils can detect latently infected cells, whether any such response is antiviral, and, if so, how latently infected cells avoid this response. Our studies show that latently infected cells can, indeed, be recognized and killed by neutrophils at high Effector to Target (E:T) ratios and that this appears to be via ADCC, which is dependent on the presence of antibodies to the viral US28 protein in HCMV-seropositive serum. However, we also show that latently infected cells inhibit recruitment of neutrophils by decreasing the expression of S100A8 and S100A9, two secreted cellular proteins that are known to mediate neutrophil recruitment. Preventing this latency-associated downregulation of S100A8/A9 resulted in increased recruitment of neutrophils to latently infected cells and their subsequent killing by ADCC.

Taken together, our work shows that myeloid cells latently infected with HCMV can be targeted and killed by neutrophils through ADCC in a US28 antibody-dependent manner. However, a latency-associated decrease in the expression and secretion of cellular S100A8/A9 prevents recruitment of neutrophils to latently infected monocytes, thereby helping to avoid neutrophil-mediated targeting and elimination of the latent HCMV reservoir.

## Results

### Neutrophils Target and Kill Latently Infected Monocytes but Only in the Presence of Serum from a Seropositive Donor

Previous analyses have shown that host T cell responses can recognize and target myeloid cells latently infected with HCMV. However, changes in the latency-associated secretome inhibit these T cell effector functions (Lau et al., 2016b, Mason et al., 2012, Mason et al., 2013). Whether other immune cells can also recognize and target latently infected cells is not known. For instance, it is becoming increasingly clear that neutrophils also target virally infected cells (Fujisawa, 2008, Jegaskanda et al., 2013, Worley et al., 2018) and, in the case of HCMV, neutrophils appear to be recruited to lytically infected cells and used to disseminate infectious virions (Pocock et al., 2017). However, it is not known if neutrophils recognize latently infected cells and, even if they did, it would be difficult to see how this could be pro-viral, as is seen during lytic infection, because no virion production occurs during latent infection (Hargett and Shenk, 2011, Poole et al., 2014a, Poole et al., 2014b). Consequently, we asked if latently infected cells are recognized by neutrophils and what effect this has on the latently infected cell.

To address this, we generated a recombinant HCMV carrying an mCherry expression cassette under the control of the cellular GATA-2 promoter (TB40E-GATA2-mCherry, detailed in the Methods section), which allowed detection of latently infected cells based on mCherry expression (Figure S1A). This virus showed no growth defects during lytic infection (Figure S1B), established latency normally (Figure S1C), produced infectious virus only after reactivation by differentiation (Figure S1D), and resulted in the detection of mCherry expressing latently infected cells for a much longer time frame compared with a recombinant SV40-GFP-HCMV (Figure S1A) in which GFP expression is known to wane after 3–4 days (Krishna et al., 2017b).

We then used this recombinant virus to analyze whether neutrophils were capable of targeting latently infected cells and what effect this would have on the cells. To this end, monocytes were latently infected with TB40E-GATA2-mCherry virus and the monocyte population, both latently infected (red) and bystander uninfected monocytes, were co-stained with calcein dye. The monocytes were then co-cultured with purified neutrophils at high E:T ratios; we used higher than physiological E:T ratios of neutrophils to monocytes (with physiological ratios in peripheral blood being approximately 1:5 monocytes:neutrophils) to overcome any potential inhibitors of neutrophil function that might have been produced by the latently infected monocytes.

**Figure 1:**
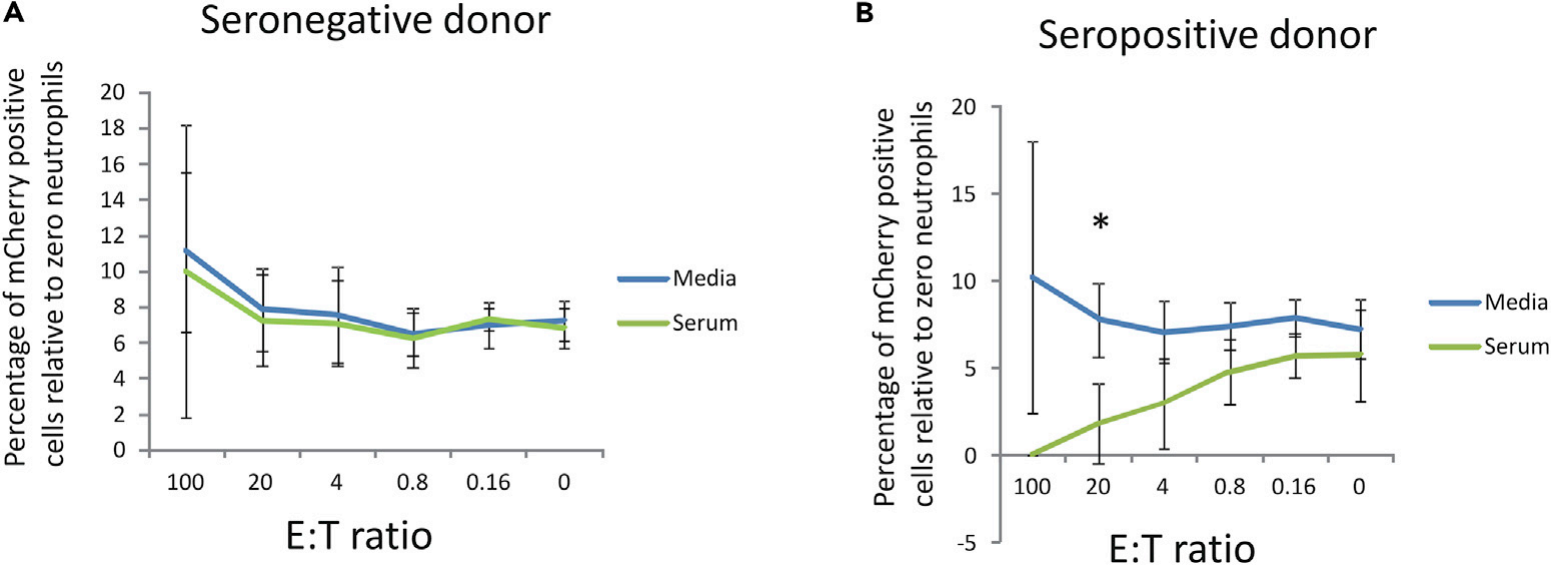
Neutrophils Decrease the Number of HCMV Latently Infected Cells at High E:T Ratios but Only in the Presence of HCMV+ Serum (A and B) CD14+ monocytes and neutrophils were isolated from an HCMV-seronegative (A) or HCMV-seropositive donor (B). These CD14+ monocytes were then latently infected for 6 days with TB40E-GATA2-mCherry. Following this, the infected monocytes were pulsed with calcein and then co-cultured with autologous neutrophils at the indicated E:T ratio in the absence (Media) or presence of autologous serum (Serum). After 6-h co-culture, the numbers of latently infected cells (red cells) were counted relative to total monocytes (green cells) by fluorescence microscopy. (A) and (B) each show data from two independent experiments (from two separate donors) using eight replicates along with standard deviation error bars.

Figure 1A shows that addition of neutrophils to experimentally latently infected monocytes from an HCMV-seronegative donor had little effect on the number of experimentally latently infected cells in the population compared with uninfected bystander cells at any E:T ratio in the presence (serum, green line) or absence (media, blue line) of serum from the same seronegative donor. However, we reasoned that, besides ROS- and NET-mediated killing, an important mechanism by which neutrophils are known to mediate cell killing of virally infected cells is by ADCC, an antibody-dependent mechanism (Sionov et al., 2015, Sips et al., 2016, Yu et al., 2016). Consequently, we repeated the analysis using monocytes and serum from an HCMV-seropositive donor (Figure 1B).

Figure 1B shows that the number of experimentally latently infected monocytes (that are mCherry positive) from an HCMV-seropositive donor are profoundly reduced, particularly at high E:T ratios of neutrophil:monocytes, when cells were co-cultured in serum from the same HCMV-seropositive donor (serum, green line). No such decrease in latently infected monocytes was observed if the analysis was carried out in the presence of media only (media, blue line). These data were consistent with the view that neutrophils are able to target and kill latently infected cells but only in the presence of serum from an HCMV-seropositive donor suggesting that this killing may be mediated via ADCC.

### Neutrophil Targeting of Latently Infected Monocytes Is Mediated by Cell-To-Cell Contacts and Results in Latently Infected Monocyte Killing

Clearly, addition of neutrophils to latently infected cells in the presence of HCMV serum from a seropositive donor resulted in decreases in the number of monocytes expressing mCherry after latent infection with TB40E-GATA2-mCherry virus. Although this was likely due to death of the latently infected cells, we wanted to rule out that this decrease was simply due to, e.g., neutrophil-mediated silencing of mCherry expression in these latently infected cells rather than their neutrophil-mediated killing.

**Figure 2:**
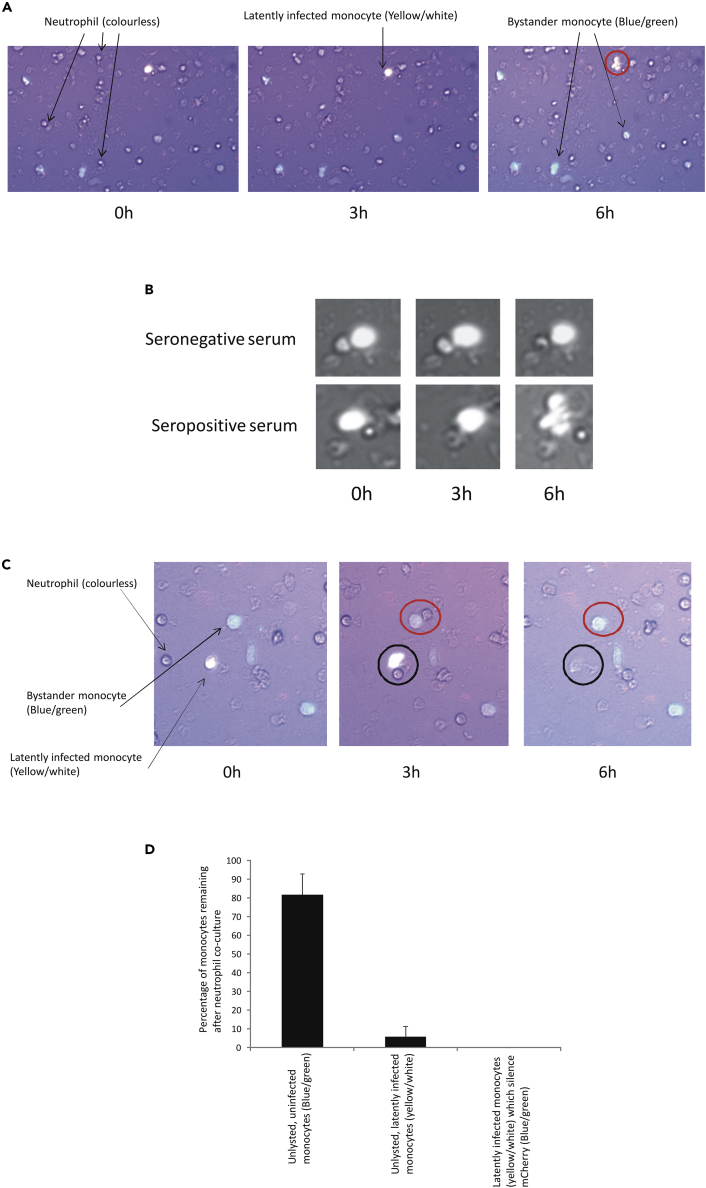
Neutrophils Mediate Lysis of Latently Infected CD14+ Monocytes in an HCMV-Seropositive Serum-Dependent Manner (A) CD14+ monocytes from an HCMV-seropositive donor were latently infected withTB40E-GATA2-mCherry for 6 days before pulsing with calcein. These cells were then co-cultured with autologous neutrophils at an E:T ratio of 20:1 in the presence of autologous serum from the same donor. Cells were then analyzed by Cellomics live-cell imaging over a time course of 6 h. In these images, calcein-positive CD14+ monocytes latently infected with TB40E-GATA2-mCherry cells appear yellow/white, neutrophils appear colorless, and uninfected bystander CD14 monocytes appear green. (B) The same as (A) except that in the top panel the CD14+ monocytes, neutrophils, and serum analyzed were from a seronegative donor (seronegative serum) and in the bottom panel, they were from a seropositive donor (seropositive serum) and cells are shown at a higher magnification to allow cell-cell contacts to be observed. (C and D) (C) Same as (A) but also shows a higher magnification to allow cell-cell contacts to be observed. Again, latently infected monocytes appear yellow/white and bystander uninfected monocytes appear green. Neutrophils are colorless. Finally, 10 neutrophils per well from 6 wells in 3 different experiments, which were observed to have contact with neutrophils, were followed by live-cell imaging, and those cells which remained intact at the end of the neutrophil co-culture were enumerated (D). In addition, 10 neutrophils from 6 wells in 3 different experiments were analyzed for changes in cell color from yellow/white (green calcein-stained plus red mCherry) to green only (whereby the GATA2mCherry signal would have been silenced) (D). The graph represents standard deviation error bars and significance determined using the Student's t test; **p < 0.001.

To do this, we employed live-cell imaging using monocytes latently infected with TB40E-GATA2-mCherry virus and co-stained with calcein such that uninfected bystander monocytes would stain blue/green (calcein positive) and mCherry-positive latently infected cells would stain yellow/white. These cultures were co-cultured for 6 h with neutrophils (unstained) at an E:T ratio of 1:20 (this E:T ratio was chosen as it gave good statistically significant neutrophil-mediated reduction of latently infected cells in Figure 1A). Figure 2A shows that when experimentally latently infected cells (mCherry) were co-cultured with neutrophils (colorless), in the presence of serum from the HCMV-seropositive donor, uninfected monocytes (green) maintained their cellular integrity as expected at all time points shown. In contrast, calcein-positive latently infected cells (yellow/white) in the same field of view (circled in the 6-h panel) clearly blebbed and died. This killing of experimentally latently infected cells, easily detectable by 6 h post co-culture, only occurred in the presence of HCMV-seropositive donor serum (Figure 2B, lower panel), but not in the presence of serum from a seronegative donor (Figure 2B, upper panel). Interestingly, killing of experimentally latently infected cells by neutrophils in the presence of HCMV-seropositive donor serum only appeared to occur upon close contact between neutrophils and the latently infected monocyte (Figure 2C, black circles 3-h and the 6-h panels). In addition, Figure 2C shows that similarly close contact between uninfected monocytes (blue/green) in the same population and neutrophils (colourless) showed no such killing (a monocyte in contact with a neutrophil is circled red in the 3-h panel, and this contact is lost as shown in the 6-h panel, where the monocyte alone is circled in red). Quantification of these data from multiple fields of view in Figure 2D shows that, of the latently infected (mCherry) and calcein-stained monocytes (yellow/white) that had contacted a neutrophil only 6% survived. However, of the uninfected monocytes in the same population (green) that contacted a neutrophil 84% survived. None of the latently infected monocytes (mCherry) and those co-stained with calcein (yellow/white) became green over time, suggesting that the decrease in the number of latently infected monocytes was due to cell death and not silencing of the mCherry cassette (Figure 2D).

Taken together, these observations suggest that, in the presence of HCMV-seropositive donor serum, neutrophils can target and kill latently infected monocytes and that this requires cell-to-cell contact between the neutrophil and the latently infected cell.

### The Requirement for HCMV-Seropositive Donor Serum in the Neutrophil Killing of Latently Infected Monocytes, in Part, Involves US28-Specific Antibodies

**Figure 3:**
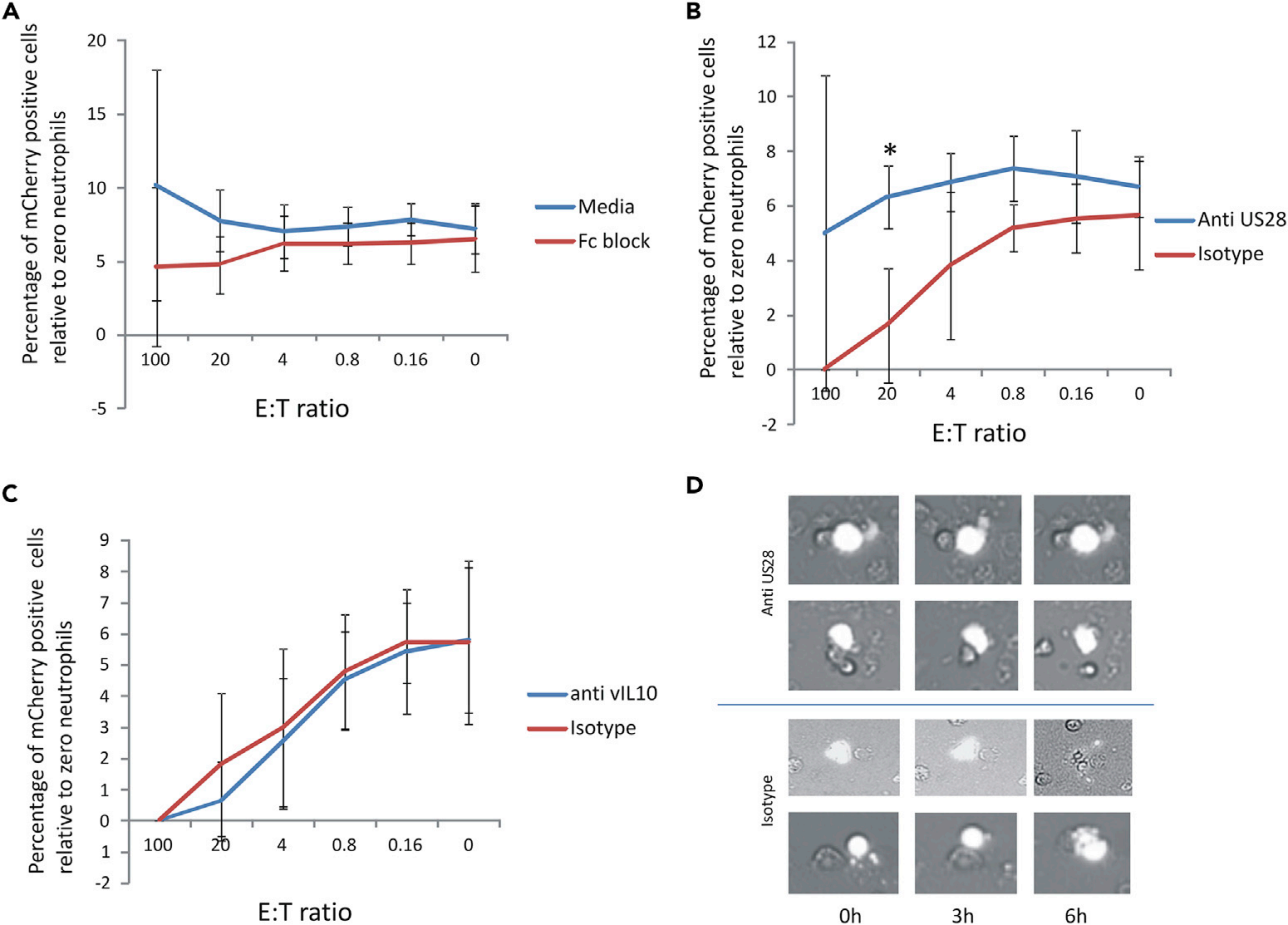
Neutrophil Killing of Latently Infected CD14+ Monocytes Is Due, At Least in Part, to ADCC Mediated by Viral Latency-Associated Protein US28 (A and B) CD14+ monocytes from an HCMV-seropositive donor were latently infected with the TB40E-GATA2-mCherry for 6 days before preincubation with media (Media) or an Fc-blocking antibody (Fc block). After this, cells were pulsed with calcein. CD14+ monocytes were then co-cultured with autologous neutrophils in the presence of serum from the same seropositive donor (red line) or media (blue line), as indicated. Data represent two independent experiments each with eight replicates. Latent monocytes (red) were enumerated relative to total monocytes (green), and standard deviation error bars are shown. (B) The same as (A) except the CD14+ monocytes were preincubated with either F(ab')_2_ isotype control antibody (isotype, red line) or an F(ab')_2_ antibody specific for US28 (anti-US28, blue line) before pulsing with calcein and co-culture with neutrophils. Data represent two independent experiments each with eight replicates. Latent monocytes (red) were enumerated relative to total monocytes (green) and standard deviation error bars are shown;***p < 0.0001. (C) The same as (B) except that a neutralizing F(ab')_2_ antibody to viral IL-10 was used (blue line) with an isotype control (red line). (D) Cells taken from the 20:1 ratio condition shown in (B) and analyzed by Cellomics live-cell imaging to show cell-cell contacts between latently infected CD14+ monocytes (yellow/white) and autologous neutrophils (colorless).

Our data, so far, had shown that the ability of neutrophils to target and kill experimentally latently infected monocytes required serum from an HCMV-seropositive donor; this suggested that the killing involved ADCC. To examine this in more detail, we first tested whether blocking all Fc receptors on the neutrophils had any impact on their ability to kill latently infected monocytes. Figure 3A shows that co-culture of latently infected monocytes with neutrophils in control media (with no HCMV-seropositive serum) showed no latently infected cell killing, as expected (media, blue line). Importantly, co-culture of latently infected monocytes with neutrophils in the presence of autologous HCMV-seropositive serum, now also showed no killing if the neutrophils were pretreated with media containing an Fc receptor block before incubation with the latently infected CD14+ monocytes (Fc block, red line) when compared with the media-only control (blue line).

As serum from an HCMV-seropositive donor and not a seronegative donor mediated killing of latently infected monocytes by neutrophils, it seemed likely that this effect was due to an HCMV-specific antibody and one that recognized antigen on the surface of latently infected cells. Although it is now becoming clear that the complexity of HCMV latency-associated gene expression is higher than first thought (Goodrum et al., 2002, Rossetto et al., 2013, Shnayder et al., 2018, Slobedman and Cheung, 2008), one well established latency-associated viral gene product, which is likely to be expressed on the surface of latently infected cells, is US28 (Avdic et al., 2016, Krishna et al., 2017b). Indeed, although the levels of US28 expression by latently infected CD14+ were variable, US28 was detectable on non-permeabilized cells (Figure S2). This confirmed cell surface expression of US28 and argued that US28-specific antibodies in HCMV-seropositive donor serum might recognize US28 on the surface of latently infected cells. To test whether recognition of this viral antigen was involved in the observed neutrophil-mediated killing of latently infected cells, we tested whether blocking of US28-specific antibodies in the serum from HCMV-seropositive donors could prevent neutrophil-mediated killing of latently infected monocytes. To do this, we removed the Fc portion from the US28-specific rabbit antibody (to prevent any potential contamination of the rabbit Fc regions of the antibodies interfering in the assay), and the resulting F(ab')_2_fragment of the US28 antibody was then used to pretreat latently infected monocytes before repeating the killing assay in the presence of serum from a seropositive donor. Figure 3B shows that when this F(ab')_2_ fragment specific to US28 was used, neutrophil-mediated killing of latently infected cells in the presence of serum from a seropositive individual was inhibited. In contrast, an F(ab')_2_ fragment from the relative isotype control had no such effect. Similarly, Figure 3C shows that if an F(ab')_2_ fragment specific to viral IL-10 (another well-established latency-associated viral gene product) was used to pretreat the latently infected monocytes, there was also no inhibition of neutrophil-mediated killing. Finally, Figure 3D shows microscopy of four representative cells from the experiment shown in Figure 3B and confirms that pretreatment of latently infected monocytes with US28-specific F(ab')2 fragment, but not the relevant isotype-matched control, prevented their killing despite being physically contacted by neutrophils.

Taken together, these data show that neutrophils can detect and kill HCMV latently infected CD14+ monocytes, at least in part, mediated by US28-specific antibody.

### Cellular Proteins S100A8/A9 Are Downregulated during HCMV Latency

**Figure 4:**
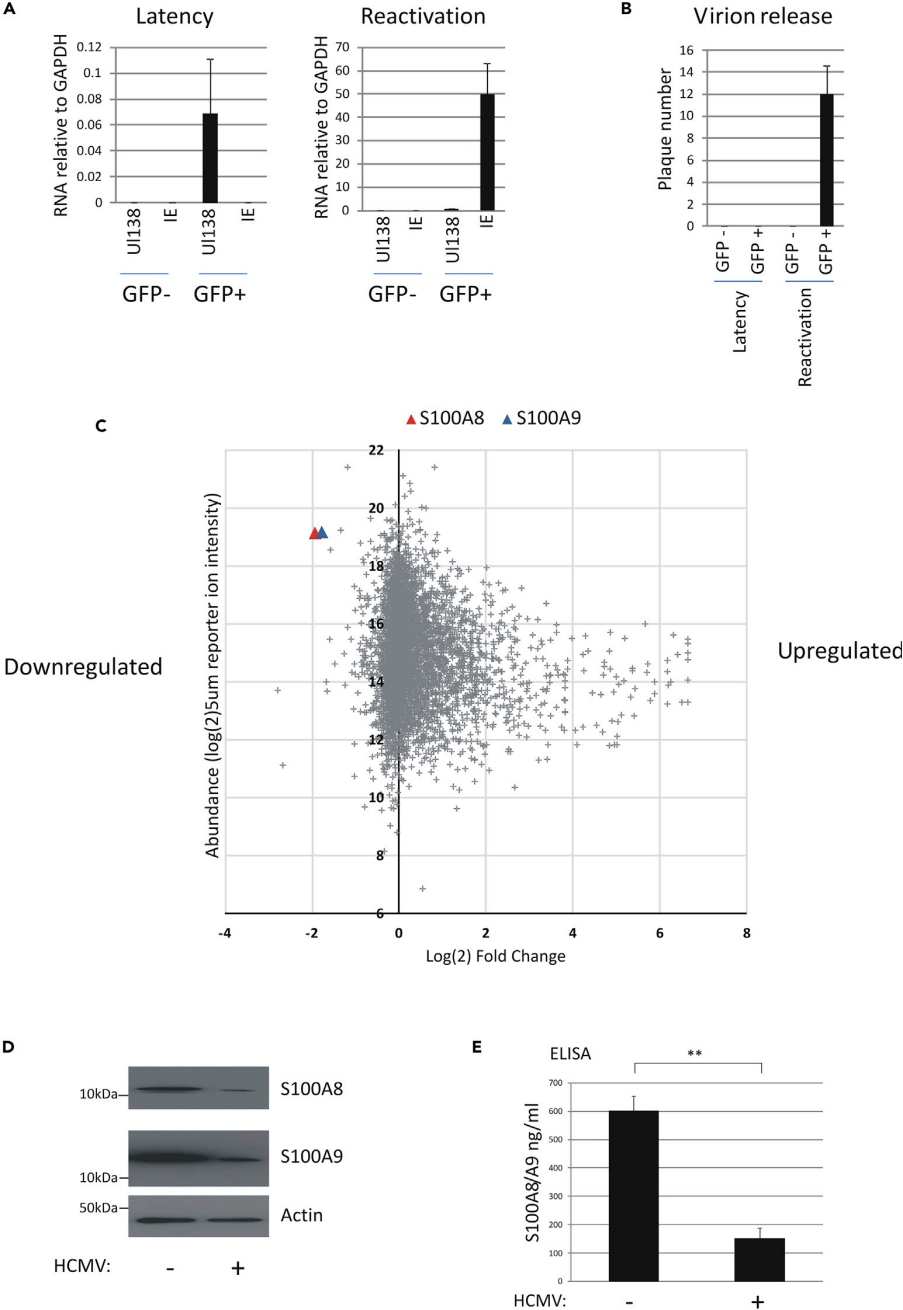
S100A8/A9 Are Downregulated during HCMV Latency (A–C) CD14+ monocytes were isolated from an apheresis cone before infecting with the SV40-GFP-TB40E isolate of HCMV. After infection, cells were washed thoroughly and cultured for 3 days in suspension before sorting into GFP-positive (GFP+) and GFP-negative (GFP−) populations. Cells were then cultured for a further 3 days following plastic adherence. On day 6 post infection, subsets of these sorted cells were cultured for an additional 6 days in X-VIVO 15 alone (latency) or in differentiation media containing IL-4/GM-CSF and LPS to reactivate virus (reactivation) and processed for RT-qPCR analysis (A). Supernatants from these cells were also transferred to indicator fibroblasts to assay for virion production (B). In all cases in (A) and (B), data shown are from triplicate samples and bars represent standard errors. Alternatively, on day 6 post-infection, the bulk of the GFP^+^ sorted cells were directly lysed and processed for total cell proteome analysis by TMT; 5,000 proteins are shown with a minimum of three unique peptides in log(2) fold changes. The S100A8 (red triangle) and A9 (blue triangle) proteins are highlighted (C). (D and E) CD14+ monocytes were also mock infected (HCMV−) or latently infected with TB40E-SV40-GFP virus (HCMV+) for 3 days before sorting and then cultured for a further 3 days before harvesting and analyzed for S100A8 and A9 proteins by western blot (D). Supernatants from (D) were further analyzed for S100A8/A9 levels by ELISA (E). Data represent two experiments of six replicates plotted with standard deviation error bars and significance determined using the Student's t test where **p < 0.001.

Thus far, our results had shown that neutrophils could kill latently infected cells but only at high E:T ratios. This suggested to us that latently infected cells may be suppressing neutrophil targeting/killing, which could be overcome by using high levels of neutrophils in the assay. To investigate this further, we decided to assess changes in total cellular protein expression during latent infection using an unbiased proteomic screen of latently infected CD14+ monocytes in an attempt to identify latency-associated changes in monocyte gene expression, which might be involved in such neutrophil evasion. Most previous studies, analyzing global changes in cellular gene expression during HCMV latency, have been carried out against a background of bystander uninfected cells (Mason et al., 2012, Poole et al., 2011, Poole et al., 2014a, Poole et al., 2015, Poole and Sinclair, 2015, Rossetto et al., 2013, Slobedman and Cheung, 2008), which can confound the identification of true latency-associated changes and their effects on cellular gene expression. To analyze global changes in cellular proteins accompanying HCMV latent infection of CD14+ monocytes, we initially enriched for the latent HCMV population by infecting monocytes with a clinical isolate of HCMV, TB40E, which expresses GFP from an SV40-GFP expression cassette, and isolated latently infected GFP-positive cells 72 h following infection (Lau et al., 2016a). GFP-positive and GFP-negative populations were cultured for a further 3 days (6 days latency, in total) before harvesting for proteome analysis. We initially confirmed that the GFP-positive population was latently infected by RT-qPCR for UL138 transcription in the absence of detectable lytic immediate early (IE) gene expression (Figure 4A). As further proof of latent infection, we also inoculated indicator fibroblasts with supernatants from latently infected cells. These fibroblasts showed no evidence of the presence of infectious virus. In contrast, supernatants from latently infected monocytes, which had been reactivated by differentiation into DCs, showed clear evidence of infectious virus production (Figure 4B). As expected, we observed no viral gene expression or virion production from the GFP-negative population (Figures 4A and 4B).

Once we had confirmed that our target cells were latent, they were lysed and proteins analyzed by tandem mass tagging (TMT) and mass spectrophotometry proteomics (Figure 4C). We compared GFP-positive and GFP-negative cells (Figure 4C), and as a control, we also compared mock infected cells with cells treated with UV-inactivated virus (Figure S3), which allowed the identification of specific changes in cellular proteins resulting from latency-associated viral gene expression rather than just interactions with virions. Total changes in cellular proteins from 2 × 10^6^ cells per condition were quantified by Mascot and Proteome Discoverer. This analysis identified the robust induction and repression of a number of cellular proteins in latently infected cells. The graph in Figure 4C represents 1,993 proteins detected (the full list of targets is shown in Table S1). This analysis resulted in the identification of a number of cellular proteins that changed as a result of latent infection of monocytes and included S100A8 and A9 (detected with 14 and 11 unique peptides, respectively), which were two of the most robustly downregulated proteins during latent infection (Figure 4C). No such downregulation of S100A8 and S100A9 was observed by infection with UV-inactivated virus when compared with mock infected cells (Figure S3).

Consistent with our proteomic analyses, western blot analysis of GFP-positive latently infected monocytes confirmed the downregulation of S100A8 and S100A9 (Figure 4D). As S100A8 and S100A9 are secreted as heterodimers (Edgeworth et al., 1991), we tested supernatants of these latently infected cells for secreted S100A8/A9 by ELISA (Figure 4E). Consistent with downregulation of both proteins in latently infected cells, a significant decrease in secreted S100A8/A9 was observed in supernatants from latently infected CD14+ monocytes. We also observed identical results if monocytes were latently infected with TB40EmCherry-GATA2 virus (Figures S1E and S1F). Together, these results show that S100A8/A9 is downregulated during latent infection with HCMV both intra- and extracellularly. Intriguingly, S100A8/A9 is a known neutrophil chemoattractant (Gomes et al., 2013, Newton and Hogg, 1998, Ryckman et al., 2003). Consequently, we reasoned that downregulation of these proteins during latency could be part of a mechanism to help the latently infected cell avoid neutrophil-mediated killing.

### S100A8/A9 Acts as a Neutrophil Chemoattractant That Is Downregulated during HCMV Latency

**Figure 5:**
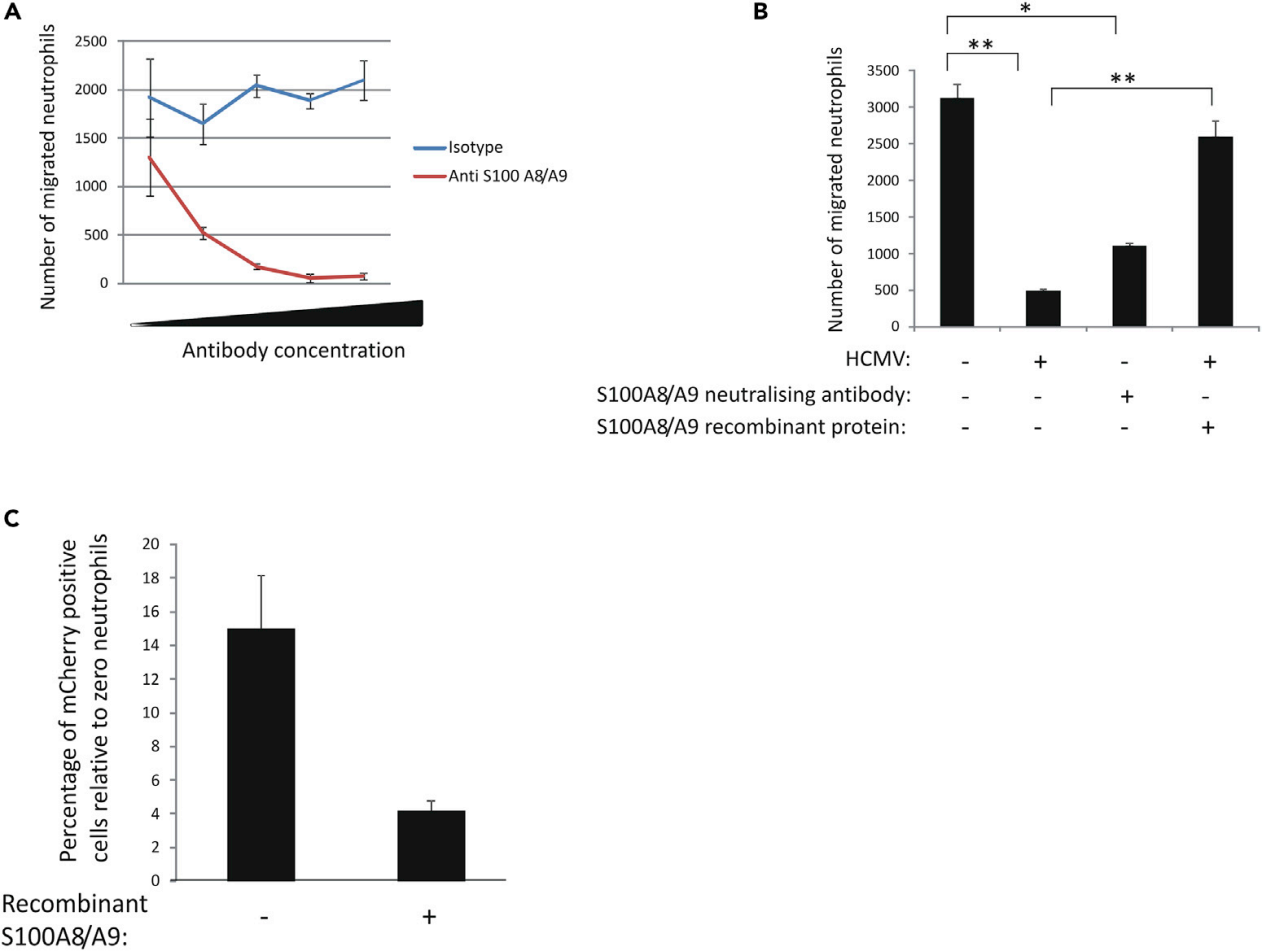
Neutrophil Migration to S100A8/A9 Can Be Blocked with Neutralizing Antibodies to These Proteins (A and B) Recombinant S100A8/A9 (500 pg/mL in cell culture medium) was incubated with increasing concentrations of isotype control antibody (blue line) or antibody specific for S100A8/A9 (red line), and this was added to the bottom of a transwell plate. Calcein-pulsed neutrophils were then added to the top of the transwell filter, and after 2 h the number of migrated neutrophils were enumerated (A). The graph shows two independent experiments with triplicate samples. Standard deviation error bars are shown. (B) Supernatants from control uninfected monocytes (HCMV-), monocytes latently infected with TB40E-GATA2-mCherry virus (HCMV+), or media supplemented (+) or not supplemented (−) with recombinant S100A8/A9 were either incubated with neutralizing antibody to S100A8/A9 (+) or the equivalent isotype control antibody (−) before placing in the bottom chamber of a transwell plate. Calcein-pulsed neutrophils were then added to the top of the transwell filter, and after 2 h migrated neutrophils were enumerated. Finally, the same experiment (as detailed in B, above) was repeated, but the monocytes were latently infected with TB40E-GATA2-mCherry virus and all monocytes pulsed with calcein and placed in the bottom of a transwell plate in the presence or absence of recombinant S100A8/A9. Neutrophils were then added to the transwells at an E:T ratio of 20. The cells were then left for 6 h before analyzing for viral cell killing relative to the absence of neutrophils (0 neutrophils). (C) The Graphs represent two independent experiments with triplicate samples. Standard deviation error bars and significance determined using the Student's t test where *p < 0.01 and **p < 0.001 are shown.

We first tested whether S100A8/A9 could act as a neutrophil chemoattractant in our system using transwell assays. These assays showed that recombinant S100A8/A9 did attract neutrophils and that this was decreased in a dose-dependent manner upon addition of neutralizing antibody to S100A8/A9 in the assays (Figure 5A). On the basis that S100A8/A9 is routinely secreted at high levels by myeloid cells, but that its secretion is decreased in latently infected monocytes, we next tested whether supernatants from latently infected CD14+ monocytes had reduced ability to recruit neutrophils compared with uninfected monocytes. To do this, CD14+ monocytes were latently infected with TB40E-SV40-GFP virus for 3 days and then GFP^+^ cells were sorted by fluorescence-activated cell sorting. These GFP^+^ cells were then cultured for a further 3 days (6 days latency) before supernatants were harvested and used to assess their ability to attract neutrophils. Figure 5B shows that, as expected, supernatants from control monocytes were capable of recruiting neutrophils, whereas this recruitment was severely impaired if supernatants from latently infected monocytes were used and this impairment could be partially rescued by the addition of recombinant S100A8/A9. In essence, the ability of monocytes to recruit neutrophils through the S100A8/A9 axis was severely compromised if these cells were latently infected with HCMV.

Finally, we asked if addition of S100A8/A9 to latently infected monocyte cultures (to replete the downregulated S100A8/A9) would overcome the low level of neutrophil recruitment to latently infected cells we had previously seen. To do this we latently infected monocytes (with TB40E-GATA2-mCherry) that had also been pulsed with calcein. These were then added to the lower wells of a transwell plate, and these cells were supplemented with recombinant S100A8/A9. We then added neutrophils to the top of the transwell plate (at an E:T ratio of neutrophils that was able to elicit killing of latently infected monocytes as shown in Figures 1 and 3). Figure 5C shows that when recombinant S100A8/A9 was added to the latently infected cells to overcome latency-associated S100A8/A9 downregulation, neutrophils now migrated to the latently infected cells, resulting in their killing. These results show that the latency-associated reduction in neutrophil migration to, and killing of, latently infected monocytes can be overcome by the addition of recombinant S100A8/A9 to the latently infected cultures.

Taken together, these data show that neutrophils are able to target and kill HCMV latently infected monocytes via ADCC. However, in culture, efficient killing only occurs at high E:T ratios as high numbers of neutrophils are required to overcome the latency-associated downregulation of the neutrophil chemoattractants S100A8/A9, which prevent neutrophil recruitment to the latently infected cell (at low E:T ratios), which results in them being less efficiently targeted.

## Discussion

It is becoming increasingly clear that latent infection of myeloid cells with HCMV is far from silent or quiescent but results in profound changes in the latently infected cell to optimize viral carriage and reactivation. It is also clear that these changes in the cell act, in part, as pro-survival signals (Poole et al., 2011, Poole et al., 2015, Poole and Sinclair, 2015, Slobedman and Cheung, 2008) as well as to prevent surveillance and targeting of the latently infected cell by host immune responses, particularly CD4+ and CD8+ T cells by regulating the latency-associated cell secretome of latently infected CD34+ cells (Mason et al., 2012). Exactly where in the host such changes in secreted cell proteins would be most effective is unclear, and there is no tractable animal model for HCMV, so *in vivo* studies are difficult. However, although we do not rule out that such latency-associated changes during latent infection could affect, e.g., CD4+ and CD8+ T cell effector functions in the periphery, we think it likely that such latency-associated changes could aid T cell evasion in, e.g., the microenvironment around latently infected cells in tissues such as bone marrow. By the same argument, we feel that latently infected CD14+ cells may also create a microenvironment in sites of latency, and we, therefore, favor the view that this likely occurs in the bone marrow or other tissue sites of latency.

The routine secretion of S100A8/A9 by monocytes (Ryckman et al., 2003 and Figure 4E) suggests that neutrophils may well be routinely chemoattracted to monocytes. Our view is that, because of this, neutrophils may well be constantly sampling potential targets but these would only be routinely killed if they were expressing recognizable signals for neutrophil-mediated killing. This would be consistent with neutrophils playing a role in routine surveillance and removal of cancerous (Challacombe et al., 2006, Di Carlo et al., 2001a, Di Carlo et al., 2001b, Matlung et al., 2018, Rajasekaran et al., 2015, Treffers et al., 2018) or virally infected cells (Sionov et al., 2015, Sips et al., 2016, Yu et al., 2016) during normal surveillance. However, downregulation of S100A8/A9 from monocytes during HCMV latency could help to reduce this neutrophil surveillance and decrease the likelihood of their killing.

The ability of a pathogen to limit its visibility to multiple branches of the innate immune system is one immune evasion strategy often employed by pathogens and, in particular, those pathogens that establish latent or persistent infections, and this also includes avoidance of neutrophil killing. ADCC-mediated killing of virally infected cells by neutrophils has been reported for a number of viruses (Ackerman et al., 2016, Ashkenazi and Kohl, 1990, Bradford et al., 1992, Chai et al., 2017, Ihara et al., 1986, Siebens et al., 1979, Smalls-Mantey et al., 2013, Veillette et al., 2015). However, except for vaccinia virus, which is known to express a protein that interferes with this (Al-Mohanna et al., 2001), little has been reported on the strategies by which other viruses evade neutrophil killing. Our results now show that HCMV employs a strategy during latent infection to prevent targeting and killing of latently infected cells by neutrophils. This neutrophil targeting is combated by the ability of latent infection to suppress the secretion of the neutrophil chemoattractants S100A8/A9, thereby preventing neutrophil attraction, which otherwise would result in high levels of neutrophil recruitment to, and killing of, latently infected cells.

The ability of latently infected monocytes to target neutrophils is likely to have far-reaching implications. Neutrophils are rapidly recruited to sites of infection or inflammation by chemotaxis where they shape the immune landscape through direct and indirect interactions with macrophages, DCs, and cells of the adaptive immune response. Although it is clear that neutrophils play a central role in the control of HCMV lytic replication (Heo et al., 2015, Yamin et al., 2016), what role, if any, neutrophils play in the context of a latent infection has so far been unclear. We now show that, at high density, neutrophils are able to detect and kill latently infected cells via ADCC by targeting US28 expression on the latently infected cell, as this killing was blocked by anti-Fc receptor antibody as well as by US28-specific antibodies. The ability of HCMV to target S100A8/A9 during latent infection may have additional roles beside evasion of neutrophil targeting. For example, it is known that S100A9 prevents DC differentiation (Cheng et al., 2008), and, therefore, any latency-associated reduction of S100A8/A9 in monocytes might modulate their responses to differentiation signals, ensuring efficient reactivation only when conditions are optimal.

It is now clear that latent infection of CD14+ monocytes results in major changes in cellular gene expression at the protein level and that this includes changes in cellular proteins that interfere with neutrophil-mediated killing of latently infected cells by decreasing their expression of the neutrophil-chemoattractant S100A8/A9. Therapies to target virus-infected cells with neutrophils are currently ongoing for HIV-1 and influenza infection (Chai et al., 2017, Stephenson et al., 2016, Wines et al., 2017). Knowing that monocytes, which carry latent HCMV, can be targets of host neutrophils, but that this is combated by latency-associated changes in the cell, opens up the possibility that this viral immune evasion mechanism during latency could, itself, be exploited by strategies to help target the latent reservoir.

### Limitations of the Study

This work shows that avoidance of neutrophil recruitment is an immune evasion strategy during experimental latency of HCMV. However, as there is no tractable animal model for HCMV latent infection the findings cannot, as yet, be verified *in vivo*.

### Ethics Statement

All human samples were obtained under ethical approval and after approval of protocols from the Cambridgeshire 2 Research Ethics Committee (REC reference 97/092) conducted in accordance with the Delcaration of Helsinki. Informed written consent was obtained from all the volunteers included in this study before providing blood samples, and all experiments were carried out in accordance with the approved guidelines.

## Methods

All methods can be found in the accompanying Transparent Methods supplemental file.

## Supplemental Information

Document S1. Transparent Methods and Figures S1–S3

Table S1. Proteome from Monocytes Latently Infected with TB40E-SV40-GFP
