# Supplementary material for: Monocytes Latently Infected with Human Cytomegalovirus Evade Neutrophil Killing
Source: iScience. 2019 Jan 8;12:13–26. doi: 10.1016/j.isci.2019.01.007 (PMC6352302; doi:10.1016/j.isci.2019.01.007)
Supplement: Document S1. Transparent Methods and Figures S1–S3 [file mmc1.pdf]

ISCI, Volume *12*

Supplemental Information

Monocytes Latently Infected with Human Cytomegalovirus Evade Neutrophil Killing

Elizabeth Elder, Benjamin Krishna, James Williamson, Yusuf Aslam, Neda Farahi, Alexander Wood, Veronika Romashova, Kate Roche, Eain Murphy, Edwin Chilvers, Paul J. Lehner, John Sinclair, and Emma Poole

TRANSPARENT METHODS, SUPPLEMENTARY FIGURES AND SUPPLEMENTARY FIGURE LEGENDS

Transparent Methods

Cells and viruses

Primary CD14+ monocytes were isolated from total peripheral blood mononuclear cells (PBMC) from apheresis cones or venous blood by MACS separation using CD14+ microbeads (Miltenyi) as described previously (Krishna et al., 2016; Mason et al., 2012). Primary neutrophils were isolated from venous blood as described previously (Farahi et al., 2017). Human foetal foreskin fibroblasts (HFFFs) were obtained from ECACC and cultured as described previously (Poole et al., 2006). All cells were incubated at 37^0C in a 5% CO_2 atmosphere unless specifically stated.

TB40E-SV40-GFP has been described previously (Krishna et al., 2016) and details of the TB40E-GATA2-mCherry cloning and validation follows later in this section.

RT-qPCR

Total RNA was isolated from cells using miRNeasy mini RNA extraction kits (Qiagen) following the manufacturer's instructions from $1x10^5$ cells in a well of a 24 well plate. Viral transcript analyses were performed using the Quantitect virus kit (Qiagen) using primers and probes as previously described (Krishna et al., 2016).

Preparation of cells carrying latent virus for cell sorting

Primary CD14+ monocytes were infected in suspension and latency established for 3 days using TB40E-SV40-GFP virus. Latently infected cells were then sorted using live cell FACS isolation using the FITC channel and then immediately washed with x-vivo 15 (Lonza) and plated on plastic for a further 3 days with media changes every 24 hours. Particulars of the proteome analysis are described below.

Transwell assays

Transwell assays were carried out using 5µm pore size transwells and allowing 2h for migration as described previously (Mason et al., 2012) except that neutrophils were pulsed with calcein (BD) and analysed for the ability to migrate.. For the neutralisation assay, 500pg/ml of recombinant S100A8/A9 (R&D) was mixed with x-vivo 15 in the presence or absence of S100A8/A9 neutralising antibody (R&D) at 200ng/ml of neutralising antibody in 5-fold decreasing concentrations of antibody

for 1 hour at RT before placing in the transwell assay. For the neutralisation of supernatants from HCMV latency, the supernatants were pre-incubated with 200ng/ml of neutralising antibody to S100A8/A9 for 1h at RT before placing in the transwell assay. The number of cells which had migrated were enumerated using Image J software with size exclusion to allow specific counting of calcein stained neutrophils.

S100A8/A9 ELISA

Detection of S100A8/A9 heterodimers in supernatant was via ELISA (BioLegend) following instructions from the manufacturer.

Neutrophil killing assay

For all analyses all cells and sera were autologous. CD14+ monocytes were isolated from venous blood and plated in 96 well plate format at 1×10^5 cells per well. Cells were then incubated overnight and either left uninfected or were latently infected with HCMV isolates, as described in the text, for 6 days. Neutrophils were then isolated from fresh venous blood obtained from the same donor 7 days after the initial bleed. Monocytes were pulsed with calcein, as described previously (Mason et al., 2012) and washed thoroughly before the neutrophils were titrated onto the monocytes at the described E:T ratios in the presence or absence of autologous serum (1:1 in x-vivo 15 media). Cells were left for 6h with or without Cellomics live cell imaging and then analysed by fluorescence microscopy and ImagePro software. For the graphical presentation, the data were analysed as follows: Initially the number of virally infected cells (as determined by red fluorescence) were counted per well in the absence of the addition of neutrophils (0 neutrophils) and worked out as a percentage relative to the total number of monocytes (stained green with calcein). This value was then taken as the 100% value (i.e. no killing), as the efficiency of virus infection can differ from independent experiment to independent experiment and donor to donor, thereby allowing a baseline of “no killing” to be determined. The level of latent cell killing by neutrophils was then determined by assessing the number of red cells relative to uninfected cells (green due to calcein pulsing) as a percentage of the number of latently infected cells in the absence of neutrophils. So, if in $1x10^4$ monocytes there were 1000 latently infected cells (i.e. 10%) in the absence of neutrophils, then if addition of neutrophils resulted in only 650 latently infected cells in a well of $9.5x10^3$ monocytes (due to e.g. some low level cell death of uninfected cells) , this would give a value of 6.8% latently infected cells. In all cases, to account for any low level non-specific killing at high levels of neutrophil addition, the value is corrected to the 0 neutrophils number.

HCMV serostatus determination

To determine the serostatus of an individual, serum was isolated from venous blood and tested by ELISA using the Capital Cytomegalovirus IgG kit (Trinity Biotech) in accordance with the manufacturer's protocol.

Immunofluorescence and western blotting

CD14+ monocytes were fixed with 4% PFA for 20 min and then blocked for 1h in 1% BSA/PBS before the addition of rabbit anti-US28 specific antibody (Source Bioscience) at a concentration of 1:50 or the equivalent isotype control in 1% BSA/PBS overnight at 4ºC. After this time cells were washed for 3 times for half an hour in PBS before staining with anti-rabbit TRITC and visualising by fluorescence microscopy and analysing with ImagePro software.

Cell lysates were analysed by SDS-PAGE and western blotting. Blots were probed with actin (Abcam) and S100A8/A9 (R&D systems) primary antibodies followed by anti-rabbit or anti-mouse HRP respectively before chemiluminescence detection with ECL reagent using the manufacturer’s instructions (Thermo Fisher).

F(ab')2 purification

F(ab')2 regions of Isotype control antibody and antibody specific for US28 (Source Bioscience) were purified from total antibody using the Pierce F(ab')2 Preparation Kit (Pierce) following the manufacturer's protocol.

Proteomic screen

<u>Lysis, digestion and clean-up of protein preps</u>

From 6 well plates, cells were washed 2x with cold PBS before scraping cells into 200uL 8Murea/50mM TEAB pH 8.5. Samples were quantified by BCA assay and 28ug of each sample was taken and adjusted to the same volume with lysis buffer. Reduction and alkylation was achieved by addition of 10mM TCEP and 20mM iodoacetamide for 20mins at room temperature in the dark followed by quenching with 10mM DTT. 3ug of rLysC (Promega) was added to each sample and incubated at 30 degrees for 3h before diluting the 1:5 with 50mM TEAB and addition of 3ug trypsin

(Proteomics Grade, Thermo Fisher Scientific) and incubation at 37 degrees overnight. Samples were acidified by adding 1 volume of 0.2% TFA and formic acid until pH was ~>2. Samples were desalted using in-house prepared microcolumns consisting of Oligo R3 resin (SCIEX) packed behind a C18 plug (Empore, 3M) in p200 pipette tips. Columns were washed with 100uL ACN and equilibrated with 2x100uL 0.1% TFA before loading of samples (3 passes over the column). Columsn were washed with 3x 50uL 0.1% TFA before sequential elution with 20uL each of 40% ACN, 70% ACN and 70% ACN with 1% FA. Samples were dried in a vacuum centrifuge.

<u>TMT Labelling</u>

Samples were resuspended in 20uL 100mM TEAB and to each tube 0.2ug of a unique TMT label for each sample was added in 8.5µL acetonitrile and incubated for 1h at room temperature. Labels were as follows: TMT reactions were quenched by addition of 3µL of 200mM ammonium formate, pooled and dried in a vacuum centrifuge. The sample was then Resuspended in 800µL 0.1% TFA and acidified to ~pH2 with formic acid before performing a C18-SPE cleanup using a Sep-Pak cartridge (Waters) attached to a vacuum manifold. C18 Eluate was dried in a vacuum centrifuge and resuspended in 40µL 200mM ammonium formate, pH10.

<u>High pH Revered Phase Fractionation</u>

Sample was injected onto an Ultimate 3000 RSLC UHPLC system (Thermo Fisher Scientific) equipped with a 2.1 i.d x25cm, 1.7µm particle Kinetix Evo C18 column (Phenomenex). Mobile phase consisted of A: 3% ACN, B:ACN and C: 200mM ammonium formate pH 10. Isocratic conditions were 90% A/10%C and C was maintained at 10% throughout the gradient elution. Separations were carried out at 45 degrees. After loading at 200µL/min for 5 mins and ramping the flow rate to 400µL/min over 5mins the gradient elution proceed as follows: 0-19% B over 10 minutes (curve 3), 19-34%B over 14.25mins (curve 5), 34-50%B over 8.75mins (curve 5), followed by a 10 min wash at 90% B. UV absorbance was monitored at 280nm and 15s fractions were collected into 96 well microplates using the integrated fraction collector. Peptide containing fractions were then orthogonally recombined into 12 fractions and dried in a vacuum centrifuge and resuspended in 10µL 5% DMSO 0.5% TFA for analysis.

<u>LC-MS analysis</u>

All samples were injected onto an Ultimate 3000 RSLC nano UHPLC equipped with a 300µm i.d. x 5mm Acclaim PepMap µ-Precolumn (Thermo Fisher Scientific) and a 75µm i.d. x50cm 2.1µm particle Acclaim PepMap RSLC analytical column. Loading solvent was 0.1% TFA, analytical solvent A: 0.1% FA and B: ACN+0.1% FA. All separations were carried out at 55 ºC. Samples were loaded at 10µL/min for

5 mins in loading solvent before beginning the analytical gradient. For High pH RP fractions a gradient of 3-5.6% B over 4 mins, 5.6 – 32%B over 162mins, followed by a 5 minute wash at 80%B and a 5 minute wash at 90%B and equilibration at 3%B for 5mins. During the gradient the Orbitrap Fusion mass spectrometer (Thermo Fisher Scientific) was set to acquire spectra.

Data Processing

All Raw files were searched by Mascot within Proteome Discoverer 2.1 (Thermo Fisher Scientific) against the Swissprot Human database and a database of common contaminants.

For TMT labelled samples the search parameters were as follows. Enzyme: Trypsin. MS1 tol: 10ppm. MS2 tol: 0.6Da. Fixed modifications: Carbamidomethyl Cysteine, TMT peptide N-termini and Lysine. Variable modification oxidised methionine. MS3 reporter ion tol: 20ppm, most confident centroid. Mascot Percolator was used to calculate PSM FDR.

Search results were further processed and filtered as follows: Peptides below a percolator FDR of 0.01% and proteins below the 0.01% protein FDR (calculated from a built in decoy database search) were rejected. Protein groups were then generated using the strict parsimony principle. Peptides both unique and razor with a co-isolation threshold of 50 and an average s/n threshold of 10 were used for quantification and a normalisation of these values to the total peptide amount in each channel was applied. Instances where a protein was identified but not quantified in all channels were rejected from further analysis. "Scaled" abundances of proteins provided by Proteome Discoverer were used to derive ratios of abundance.

Cellomics live cell imaging

Live cells were automatically imaged and analysed using Thermo Scientific Array Scan (formerly Cellomics) XTI High Throughput Screening Microscope equipped with Live Cell Module, Objective Module, CCD Camera, solid-state 7 colour LED light engine providing 386 nm, 438 nm, 485 nm, 549 nm, 560 nm, 650 nm and 740 nm excitation wavelengths and 5 or 6 position emission filter wheel for Widefield fluorescence detection and a Brightfield Module with a single colour LED light source. To maintain cell health throughout, all imaging experiments the Array Scan plate acquisition chamber temperature and CO_2 level were kept at 37ºC and 5% retrospectively using Live Cell Module.
All images were acquired with Zeiss 20x/0.4 Korr LD Plan NEOFLUAR objective and X1 highly sensitive CCD camera with 4.5 micron/pixel resolution. The X1 camera was set to 2x2 binning mode. The instrument is supplied with Thermo Scientific HCS Studio Cell Analysis Software for further cellular image quantitative analysis.

For fluorescence detection 5 multiband wavelength dichroic and emitter filter set was used. GFP (or Cell Track Green dye) and mCherry signals from infected cells were detected in 485-20 BGRFRN (BGFRN states for Blue, Green, Red, Far Red and Near-IR emission) and 560-25 BGRFRN channels retrospectively. White light images were obtained in BGRFRN – BRIGHTFIELD channel. Cells were acquired simultaneously in all channels. The cells were cultured, treated and imaged in 96 well micro plates. The entire area of each well for all 96 wells in all experiments was imaged to acquire a statistically significant amount of a low frequency of GFP expressing cells for future analysis.

Generation of TB40E-GATA2-mCherry virus

TB40E-GATA2-mCherry was generated by replacing the SV40-GFP cassette with a GATA2-mCherry cassette on the basis that latency-associated gene promoters are rich in GATA-2 binding sites and depend on GATA-2 in the absence of lytic IE72 expression (Poole et al., 2013). To generate TB40E-GATA2-mCherry virus gBLOCKs from IDT (Coralville, IA) were utilised with primers for recombineering using the GalK/2-DOG selection method. The GATA promoter has high GC content therefore Phusion Polymerase was used with standard protocols from the manufacturer in proprietary buffer (Phusion GC buffer). The GalK insertion used the following two primers to generate the insertion construct with the pGalK plasmid as a PCR template:

GATA galk ins 5’
TGG GGA TGA AAT ATA TCC AGA TAC GCA GTT TTG TTA TCC TAA CAA AAC CCG TGT CAT GCC CTG TTG ACA ATT AAT CAT CGG CA
GATA galk ins 3’
ACC AGG ATG GGC ACC ACC CCG GTG AAC AGC TCC TCG CCC TTG CTC ACC ATG GTG TCA GCA CTG TCC TGC TCC TT

Excision of GalK (by 2-DOG selection) was achieved with a PCR product made using the GATA2promoter gBLOCK as template and using the following 2 primers:

GATA galk reversion 5'
TGT CGC ATA AAA GGC GGT GGG ATG TGG GGA TGA AAT ATA TCC AGA TAC GCA GTT T
GATA galK reversion 3'
GCC GTT TAC GTC GCC GTC CAG CTC GAC CAG GAT GGG CAC CAC CCC GGT G

Supplementary figures

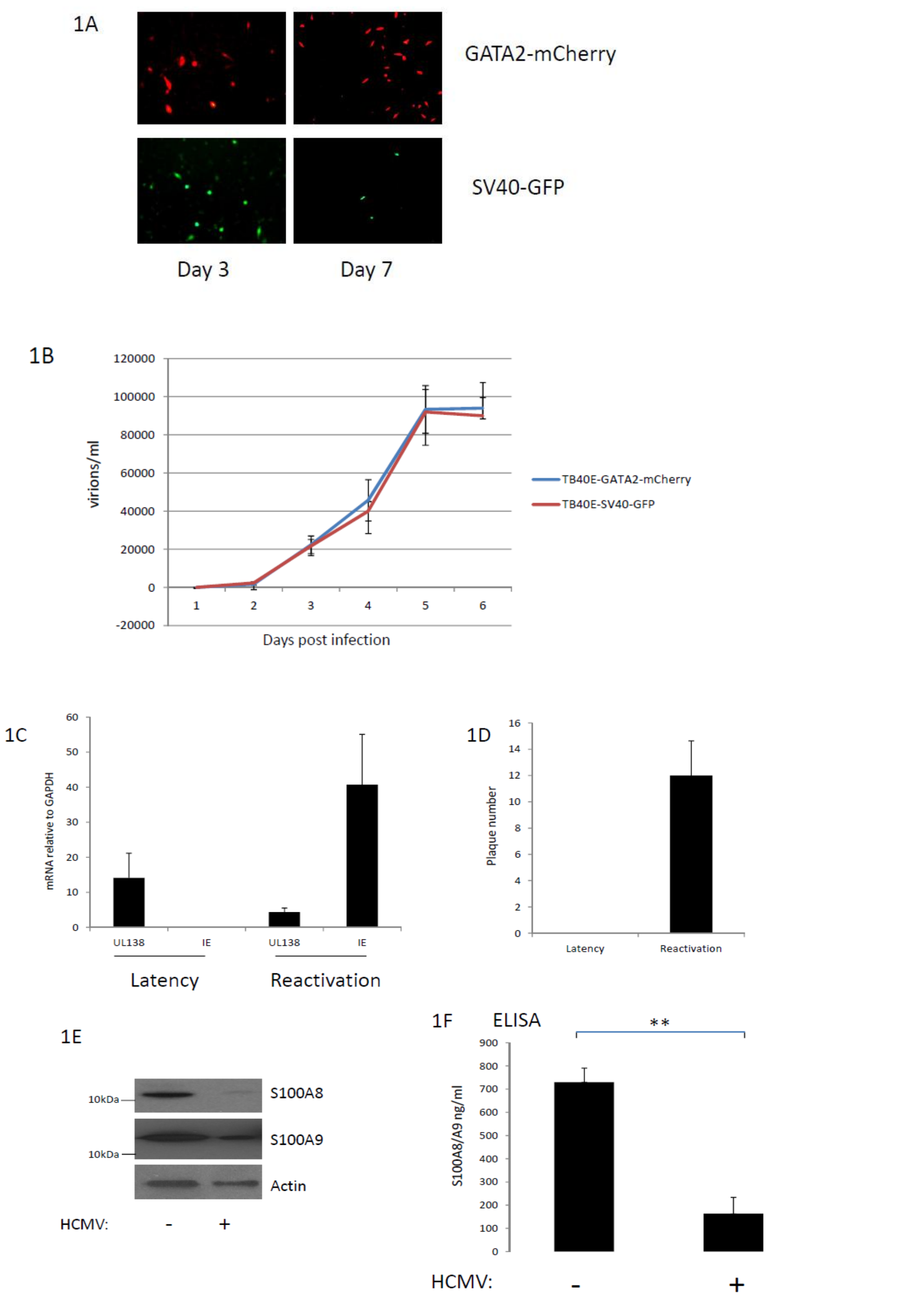

Figure S1 relates to all figures throughout the pape**r. Characterisation of the TB40E-GATA2-mCherry virus.** (A) CD14+ monocytes were latently infected with TB40E-SV40-GFP virus (SV40-GFP) or TB40E-

GATA2-mCherry (GATA2-mCherry) virus for 3 and 7 days. (B) HFF cells were infected with either TB40E-SV40-GFP or TB40E-GATA2-mCherry and supernatants from 1-6 days post-infection were analysed for infectious virus production by inoculation of fresh fibroblasts followed by indirect immunofluorescence staining for IE1 positive cells allowed numbers of infectious units/mL of supernatants to be quantified. (C) CD14+ monocytes latently infected with TB40E-GATA2-mCherry were analysed by RTqPCR analysis for cellular GAPDH, UL138 or IE RNA (Latency) as well as after their differentiation and into mature dendritic cells with GM-CSF/IL-4 and LPS (reactivation). (D) Supernatants from these cells were also transferred onto indicator fibroblasts to test for virus production. (E) CD14+ monocytes were latently infected for 3 days with TB40E-GATA2-mCherry virus (HCMV+) or mock infected (HCMV-), sorted according to mCherry expression and then cultured for a further 3 days (6 days latency) before harvesting and analysis by western blot for S100A8, S100A9 or actin. (F) Supernatants from (E) were also analysed for S100A8/A9 by ELISA. Data represent two independent experiments each with 6 replicates plotted with standard deviation error bars and significance determined using the student's T test where **=$p<0.001$

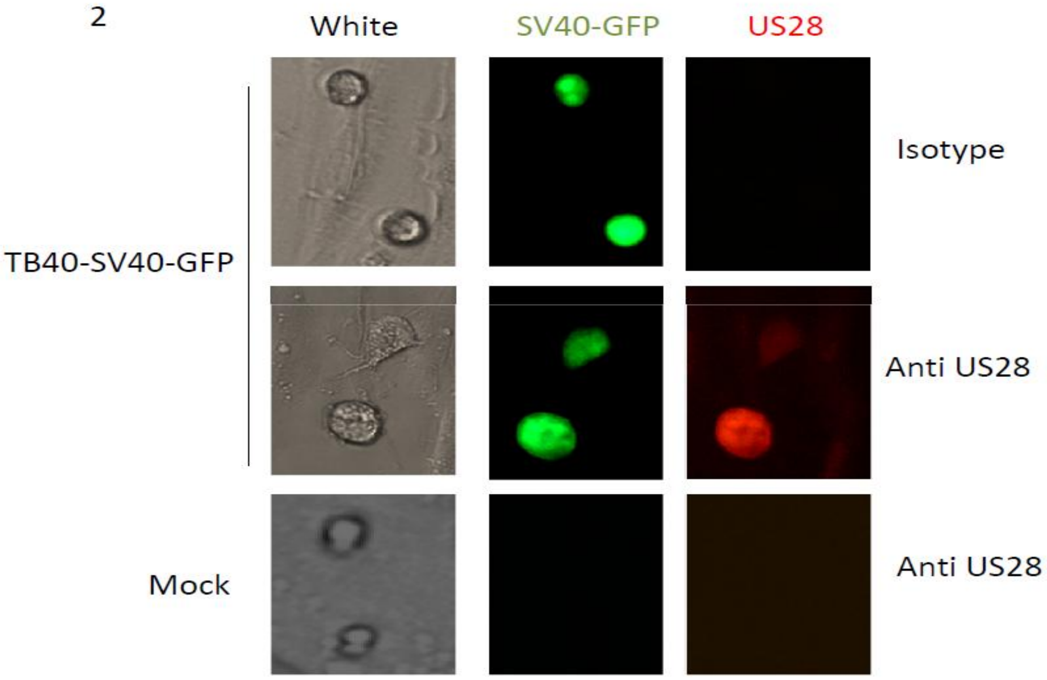

Figure S2 relates to figure 3 in the paper. **US28 antibody detects US28 during on the surface of latently infected CD14+ monocytes.** CD14+ monocytes were left uninfected (mock) or latently infected for 6 day with TB40E-SV40-GFP (SV40-GFP) before fixing and then staining with anti-US28 antibody or an isotype matched control antibody.

3

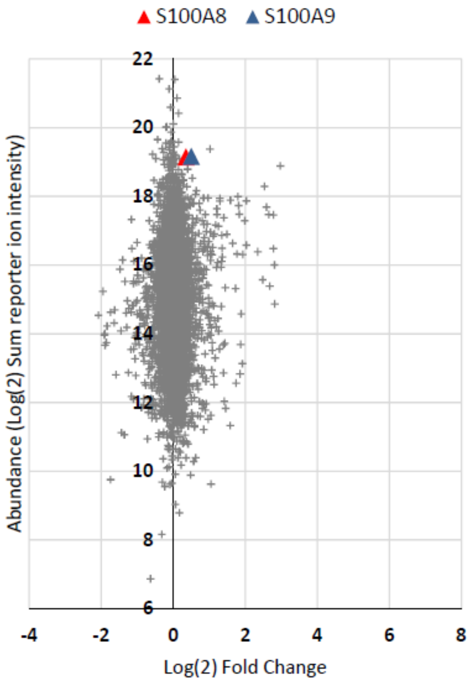

Figure S3 relates to figure 4 in the paper. **UV inactivated virus has no effect on S100A8/9 levels.** Monocytes were either untreated or infected with uv-inactivated virus. On day six post-infection, the cells were directly lysed and processed for total cell proteome analysis by Tandem Mass Tagging (TMT); 5000 proteins are shown with a minimum of 3 unique peptides in Log(2) fold-changes. The S100A8 and A9 proteins are highlighted (triangles).
